# Oncogenic super-enhancer formation in tumorigenesis and its molecular mechanisms

**DOI:** 10.1038/s12276-020-0428-7

**Published:** 2020-05-07

**Authors:** Qunying Jia, Shuhua Chen, Yuan Tan, Yuejin Li, Faqing Tang

**Affiliations:** 1 Hunan Key Laboratory of Oncotarget Gene and Clinical Laboratory, Hunan Cancer Hospital and the Affiliated Cancer Hospital of Xiangya School of Medicine, Central South University, Changsha, 410013 China; 2 Department of Otolaryngology, The Second People’s Hospital of Foshan, Foshan, 528000 Guangdong China

**Keywords:** Oncogenes, Oncogenes, Oncogenesis

## Abstract

Super-enhancers (SEs) consist of a cluster of many enhancers bound to a great number of transcription factors. They are critical cis-regulatory elements that determine the identity of various human cell types. During tumorigenesis, DNA mutations and indels, chromosomal rearrangements, three-dimensional chromatin structural changes, and viral infections mediate oncogenic SE activation, and activated SEs have been found to regulate the expression of oncogenic genes. Inhibition specifically targeted to oncogenic SE assembly and activation provides a novel powerful therapeutic strategy for various cancers. In this paper, we first introduce the current understanding of oncogenic SE assembly and activation and then summarize the pathogenic factors and mechanism of oncogenic SE activation. Next, we elaborate on the oncogenic functions of SEs in cancers and the application of SEs as therapeutic targets. Finally, we turn our focus to the use of SEs in basic research and clinical trials.

## Cancer: Enhancing the expression of cancer-causing genes

Drugs that block the assembly and activation of large DNA segments involved in enhancing gene expression could help in the treatment of cancer. Faqing Tang of Hunan Cancer Hospital in Changsha, China, and colleagues review the ways in which cancer cells hijack clusters of gene-regulating sequences known as super-enhancers, regulatory gene regions that normally help determine a cell’s unique identity, to drive the aberrant gene activity that fuels tumor growth. The researchers describe how numerous factors, ranging from internal DNA alterations, both large and small, to viral infections and other external assaults, can spur the formation of cancer-causing super-enhancers, leading to out-of-control gene expression. Therapies that selectively target these super-enhancers are now in early clinical testing. However, more studies of super-enhancers and their role in cancer development are needed to inform future drug development.

## Introduction

The enhancer was first defined as a short DNA sequence from the Simian virus 40 genome that had a great ability to enhance the transcription of its target genes in mammalian cells^1^. Since their discovery, enhancers have been increasingly studied, with a number of enhancers identified and their structure and regulatory mechanism extensively clarified^2^. Transcription factors (TFs) bind to enhancers to recruit coactivators such as the mediator complex CREB-binding protein (CBP) and p300 to alter the chromatin spatial structure, resulting in the interaction of TFs with enhancers, promoters or RNA polymerase^2^. Epigenetic modifications to histones and DNA have been proven to be the main mediators of enhancer editing and maintenance. Histone modification is related to the active state of the enhancer; for example, monomethylation of histone H3 protein at lysine 4 (H3K4me1) and acetylation at lysine 27 (H3K27ac) are correlated with functional enhancers^3^. Generally, enhancers are short noncoding DNA segments. They can be recognized by TFs and activate transcription independent of enhancer position or orientation in the genome^1^. Enhancers are transcribed into enhancer RNAs (eRNAs), the expression level of which is associated with the expression of genes nearby proximal enhancers, suggesting that enhancers play important roles in gene transcription^4^.

**Fig. 1:**
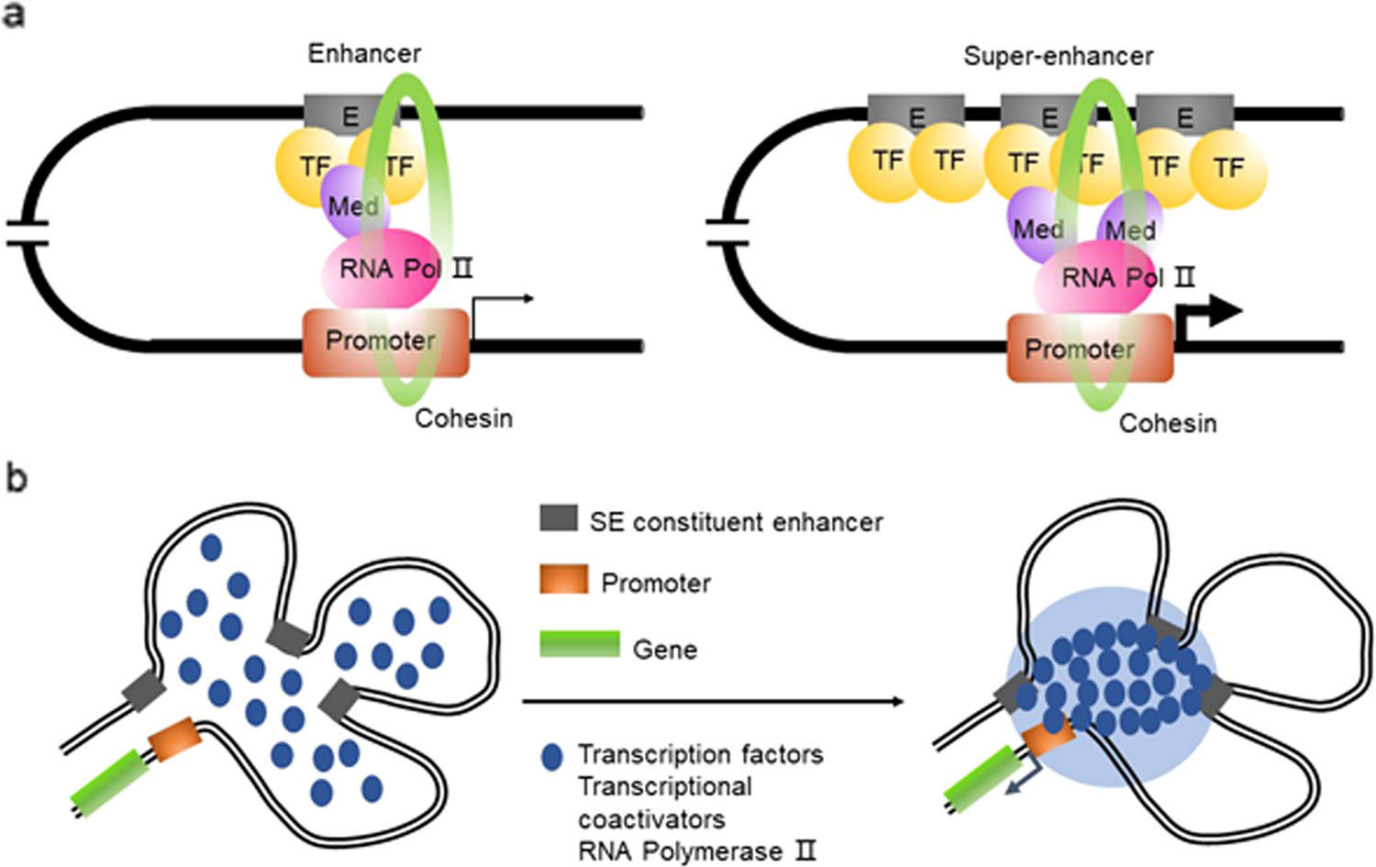
Schematic illustration of SE function and activation. **a** Enhancers and SEs are occupied by a high density of transcriptional regulators, including transcription factors, coactivators, and the RNA pol II complex. **b** A phase separation model of SE activation. High-density interactions between transcriptional regulators form phase-separated multimolecular complexes at the SE locus, leading to the transcription of SE-driven genes.

In addition to the typical enhancers, there is another type of enhancer called super-enhancers (SE) or stretch enhancers, which frequently span several kilobases (averaging approximately 9 kb in length). SEs bind with abundant tissue-specific TFs in various cells and master TFs, such as OCT4, SOX2, and Nanog, in embryonic stem cells^5,6^. Similar to typical enhancers, SEs are occupied by TFs, mediator complexes, chromatin regulators and the RNA polymerase II (pol II) complex, but the density of these active molecules on SEs is several-fold that of typical enhancers. As a result, SEs can drive targeted gene transcription more dramatically than typical enhancers^5^ (Fig. 1a). Moreover, SEs produce a higher level of eRNA (called seRNA) than is produced by a typical enhancer^6^. seRNA has extensive and far-reaching significance in physiological, biochemical, and pathological processes^7^. SEs exhibit similar action mechanisms to typical enhancers. The interaction of an enhancer or RNA pol II with a promoter is facilitated by an enhancer loaded with a cognate promoter to form a loop structure, and then, the basal RNA pol II transcription machinery is recruited to the promoter to initiate downstream transcription^8^. SEs additively and synergistically influence each other, and constituent enhancers demonstrate a temporal and functional hierarchy^9–11^. Recently, increasing evidence has revealed that SEs play vital roles in tumorigenesis and that SEs may be promising therapeutic targets for tumor treatment^5^. During tumorigenesis, DNA mutations and indels^12^, chromosomal rearrangements^13–15^, three-dimensional (3D) chromatin structural changes^16,17^, and viral infections^18,19^ mediate the generation of oncogenic SEs that drive oncogene transcription in the cells that acquire them^15,20–22^. In this paper, we elaborate on SE structure and activation modes, introduce SE regulatory mechanisms and explain their roles in the initiation and development of tumors, and discuss therapeutic strategies targeting oncogenic SEs.

## SE characteristics and identification

An SE is significantly different from a typical enhancer (Fig. 1a). Currently, an SE is defined as a cluster of enhancers that spans a large region of the genome with a median size of 8.7 kb^5^. The components associated with enhancer activity, such as mediator complexes, chromatin factors, H3K27ac and H3K4me1, histone acetyltransferases, p300 and CBP histone acetyltransferases, RNA pol II, and eRNA, are enriched in association with SEs and exhibit increased chromatin accessibility^6^. SEs are characterized by the differential binding of tissue-specific TFs. The levels of master TFs such as OCT4, SOX2, and Nanog are similar for typical enhancers and SEs, while Klf4 and Esrrb occupy SEs at significantly higher rates than they occupy typical enhancers^5,6^. Compared to typical enhancers, SEs are more frequently bound by terminal transcription factors in the Wnt, TGF-β, and leukemia-inhibitory factor (LIF) signaling pathways, and SE-driven genes are much more sensitive than typical enhancer-driven genes to perturbations in associated enhancer-binding transcriptional regulator genes^5,11,21–23^. Compared with typical enhancers, individual constituent enhancers of SEs are capable of increasing transcriptional activation levels^5^. Some evidence indicates that constituent enhancers within an SE interact with each other additively or synergistically and have nonredundant functions in gene regulation^9–11^, while the deletion of constituent enhancers may compromise the activity of other SE components^9,11^, leading to dysfunction of the entire SE^12^.

The formation of an SE is proposed in a schematic model (Fig. 1a). SEs have a high number of binding sites for TFs to which MEDs are recruited to alter the chromatin spatial structure, resulting in the interaction of TFs with enhancers, promoters, or RNA pol^24^ (Fig. 1a). A phase separation model has been proposed to clarify the mechanisms underlying the formation, function, and properties of SEs^24,25^ (Fig. 1b). Through a phase separation phenomenon similar to polymer condensation, heterogeneous mixtures of proteins and nucleic acids are assembled into membrane-less organelle structures. Phase-separated biomolecule condensation is a mechanism by which biochemical reactions are compartmentalized and concentrated in cells^26^. These membraneless organelles rapidly exchange components within the cellular milieu, which is readily altered in response to environmental cues. Dynamic, synergistic, and multivalent intermolecular interactions are associated with liquid–liquid phase separation^27^. A recent study showed that intrinsically disordered regions (IDRs) of BRD4 and MED1 can form phase-separated droplets at sites of SE-mediated transcription, and MED1-IDR droplets can compartmentalize and concentrate the transcription apparatus to maintain their separation from nuclear extracts. Thus, it is speculated that SE condensates facilitate the compartmentalization and concentration of the transcriptional components at specific genes through the phase-separating properties of the IDRs in the TFs and cofactors^25^. Initiation of phase-separated condensate formation has also been associated with the activation domains in the master TFs OCT4 and GCN4 and mediator complexes^28^. The absence of cohesin leads to the extensive fusion of SEs in the nucleus, which has been implicated in constraining SE–SE interactions^29^. These reports provide a new model for elucidating transcriptional regulation and explaining the different aspects of SE biology^24^.

In SE identification, high-throughput sequencing and next-generation sequencing (NGS) technologies provide particularly powerful tools for genome-wide identification and enhancer/SE prediction. These approaches are primarily based on chromatin immunoprecipitation followed by high-throughput sequencing (ChIP-seq)^30^, DNase I coupled to high-throughput sequencing (DNase-seq)^31^, and chromosome conformation capture (3C)^32^. On the basis of these tools, a series of derived methods, such as ChIP-exo^33^, FAIRE-seq^34^, GRO-seq^35^, ChIA-PET^36^, ATAC-seq^37^, STARR-seq^38^, Hi-C (chromosome conformation capture coupled to sequencing)^39^, and HiChIP^40^, have also been applied to identify enhancers/SEs. ChIP-seq is a high-resolution, low-noise, and high-coverage research method for the genome-wide analysis of histone modification, nucleosome localization, and the distribution of transcription factor-binding sites^30^. ChIP-seq uses histone modification marks to identify molecules presumably associated with SEs, such as transcription factors, the transcription cofactor p300, and the H3K27ac and H3K4me1 histone modifications^5,6,30^. DNase-seq is a biotechnology that uses high-throughput sequencing technology to analyze DNase I hypersensitive sites in enzyme-sensitive regions for enhancer prediction^31^. The analysis of the mediator cohesin was carried out by ChIP-seq, and the interaction between chromatin can be directly analyzed by 3C, 4C (circularized chromosome conformation capture), 5C (chromosome conformation capture carbon copy), or Hi-C technology, with the genes related to enhancers determined at the same time^13,32^. The identification of genome-wide enhancers/SEs enables a more systematic and comprehensive study of enhancers/SEs in biological processes.

## Functions of oncogenic SEs

During tumorigenesis, tumor cells acquire specific SEs to promote oncogene expression, which mediates the dysregulation of signaling pathways^6,12,22,41^. These specific SEs are known as oncogenic SEs. Oncogenic SEs were first identified in multiple myeloma cells and bind at high density to MED1 and BRD4^21^. H3K27ac ChIP-seq data have been used to identify SEs in 18 human cancer cells, including cells from colorectal, prostate, pancreatic, breast, lung, liver, and cervical cancers and multiple myeloma, CML, T cell leukemia, lymphocytic leukemia, and glioblastoma^6^. In recent years, several oncogenic SEs have been found in various cancers, including neuroblastoma, small-cell lung cancer, medulloblastoma, esophageal cancer, gastric cancers, and melanoma^42^.

Oncogenic SEs promote cell malignancy by increasing oncogene transcription^11^. Mechanistically, oncogenic SEs may activate the MAPK signaling pathway to inhibit apoptosis and increase cell proliferation^43^. SEs also mediate the overexpression of the v-ets erythroblastosis virus E26 oncogene homolog (ERG), resulting in target gene expression to promote cancer development^44^. In addition, oncogenic SEs increase the expression of CYP24A1, GJA5, SLAMF7, and ETV1^45^. Nucleus translocation of SEs increases MYB expression in adenoid cystic carcinoma (ACC), and SEs promote the expression of TERT in pheochromocytomas and paragangliomas^46^. CRC-associated SEs are enriched at transcription factor 4 (TCF4) binding sites^11^. ChIP-seq analysis of CRC cells showed that TCF4 is a terminal TF in the Wnt pathway and occupies the c-MYC locus. TCF4 is a target of Wnt signaling that shows a strong H3K27Ac signal after cancer cells acquire oncogenic SEs^11^. ChIP-seq analysis of H3K27Ac in MCF-7 cells indicated that the SE-targeted *ESR1* gene encodes only estrogen receptor alpha (ERα); furthermore, this oncogenic transcription factor can distinguish cancer subtypes through distinct signaling pathways. In ER-positive breast cancer cells, SE-targeted genes are enriched in processes involved in ERα binding, whereas in triple-negative breast cancer cells, the SE-enriched sites are different from those enriched by oncogenic TFs^6,47^. The general function of SEs may involve channeling oncogenic signaling pathways into gene expression programs that are required for sustaining cancer development^11^.

## Formation of oncogenic SEs

A large number of genome-wide studies have revealed that disease-related somatic variations occur mainly in noncoding genomes and are often enriched in regulatory regions^48,49^. Germline and somatic cells appear to acquire SEs through various mechanisms, including genomic deletions, duplications, translocations, insertions, inversions, and single-nucleotide polymorphisms (SNPs). These genetic alterations can disrupt TF-binding sites in putative SEs, modify SE copy number, and change the genomic space, which lead to SE activation or inhibition, ultimately resulting in the deregulation of nearby target genes^13,16^. In summary, novel oncogenic SEs may originate through a variety of mechanisms, including (1) mutations and genomic alterations^12,50–52^, (2) chromosomal rearrangements^14,15,23,41,53–55^, (3) spatial alterations in SE location by 3D chromatin structural changes^16,17^, and (4) viral oncogenes^18,19,56,57^.

### DNA mutations and indels result in the formation of oncogenic SEs

**Fig. 2:**
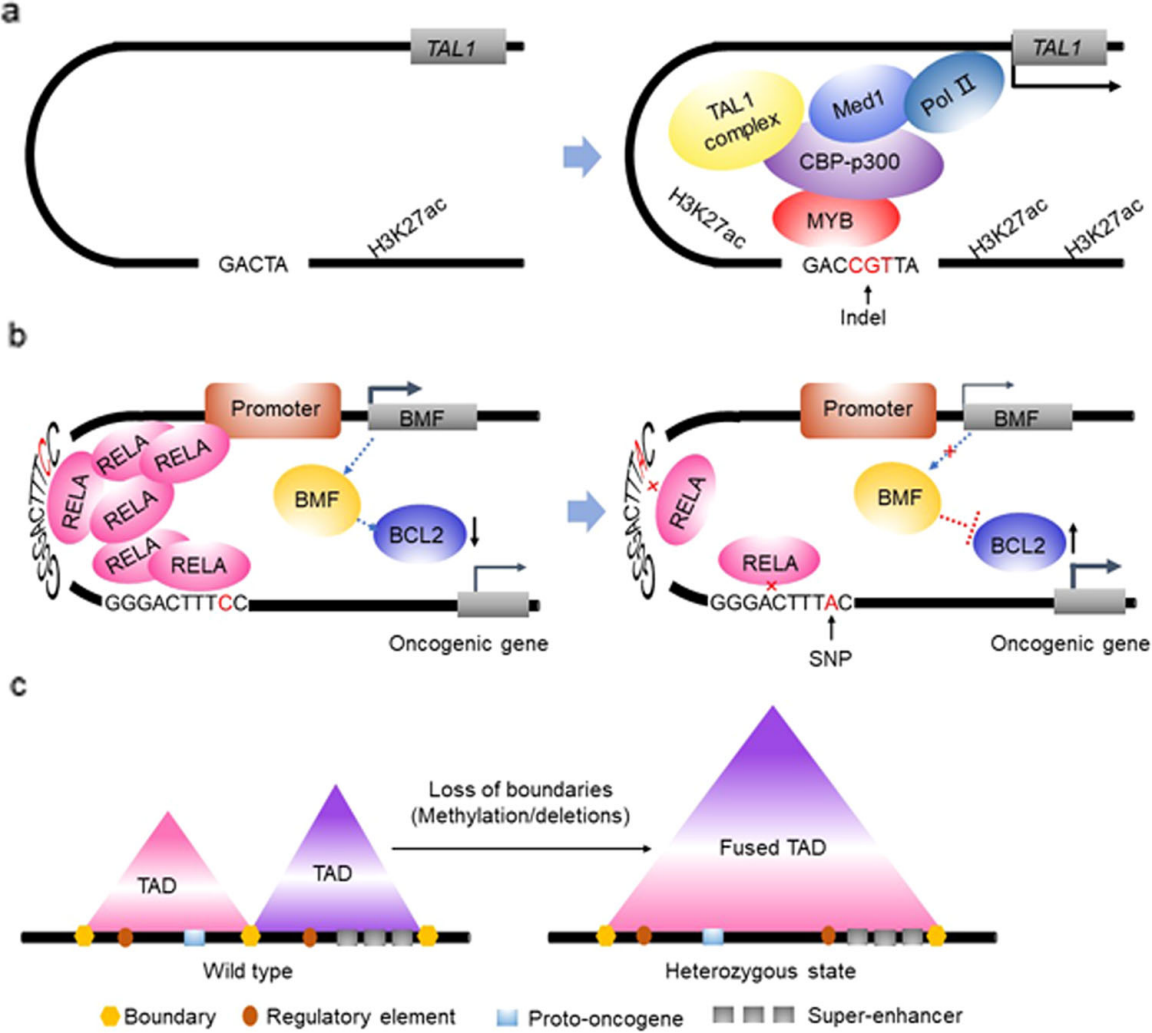
Various mechanisms of oncogenic SE formation. **a** Small insertions in the noncoding intergenic region upstream of the *TAL1* oncogene induce de novo binding sites for the TF MYB, leading to the formation of SEs that drive TAL1 expression. MYB binds and recruits its H3K27 acetylase binding partner CBP, the TAL1 transcriptional complex containing RUNX1 and GATA-3. **b** The SNPs in the 15q15.1 risk locus generate SEs for the proapoptotic gene *BMF* and disrupt the binding of the TF RELA to SEs, leading to activation of the antiapoptotic function of BCL2 and promoting tumorigenesis. **c** Oncogene activation occurs *via* structural variations or epigenetic deregulation.

The sequences comprising enhancer/SE DNA are mutated to alter promoter and enhancer/SE function. In T cell acute lymphoblastic leukemia (T-ALL), small insertions of 2–18 bp in the noncoding intergenic region upstream of the *TAL1* oncogene produce de novo binding sites for the transcription factor MYB, resulting in SE formation to drive TAL1 expression^12^. Binding to these de novo sites, MYB recruits CBP/p300 acetyltransferase and TAL1 transcription factor complexes to promote the formation of oncogenic SEs and drive key gene expression in leukemogenesis (Fig. 2a). In addition to small insertions, SNPs are often found to initiate the activity of an oncogenic SE. For example, in neuroblastoma cells, the formation of an SE at the *LMO1* oncogene locus is dependent on the binding of GATA3 to a conserved GATA-binding site. An SNP located near the SE alters a conserved GATA-binding motif, changing it to a TATA motif, which results in a significant reduction in SE activity and LMO1 expression^52^. In addition, SNPs disrupt SEs associated with tumor suppressor genes to promote tumorigenesis. A meta-analysis of genome-wide association studies showed that the 15q15.1 risk locus of the *BMF* (BCL2-modifying factor) gene carries chronic lymphocytic leukemia (CLL) susceptibility. The SNP in the 15q15.1 risk locus generates SEs to regulate the proapoptotic gene *BMF* and disrupts the binding of the TF RELA to the SE, leading to an increase in BCL2 antiapoptotic function and the promotion of tumorigenesis^51^ (Fig. 2b).

### Chromosomal rearrangements generate oncogenic SEs

Genomic rearrangements, inversions, translocations, and deletions move SEs from their natural genomic context to oncogene regions, leading to SE activation. This phenomenon is known as “Super-enhancer hijacking” and has been reported in various cancers, including acute myeloid leukemia (AML), neuroblastoma, medulloblastoma, and colorectal cancer^13–15,41,53^. One classic example is the inversion of a 9-kb fragment in AML cells that redirects an SE from its role as a *GATA2* tumor suppressor to an *EV1* oncogene enhancer, leading to the downregulation of tumor suppressors and oncogene activation^15^. Another example of enhancer hijacking was observed in ACC, a chromosomal translocation repositioning a distal SE to a location proximal to the *MYB* gene, leading to high MYB expression^55^. Further 3C analysis confirmed chromatin interactions between the MYB promoter and the aberrantly translocated SE. Furthermore, the translocated SE element was found to contain MYB-binding sites, which were actively bound by MYB itself to form a positive feedback loop, further enhancing MYB expression. Most of the samples from a subgroup of medulloblastomas, especially those with highly expressed growth factor independent 1 family proto-oncogenes (*GFI1* and *GFI1B*), had recurrent structural variations that resulted in the relocation of *GFI1* and *GFI1B* into close proximity of foci occupied by active SEs, initiating oncogenic activity^41^. A recent example of enhancer hijacking was that of hybrid SEs generated by *C19MC*–*TTYH1* gene fusions that amplified the *C19MC*–LIN28A–MYCN oncogenic circuit and drove the expression of embryo-restricted DNMT3B6 to promote a primitive malignant epigenetic state in embryonal tumors with multilayered rosettes^58^. In addition to genomic rearrangements, copy number variations can also result in oncogenic SE activation. Somatic copy number and tissue-specific epigenetic analyses of 12 cancer cell types showed that focal amplification of SEs near *KLF5, USP12, PARD6B, MYC*, and other cancer-related genes could drive aberrant expression of oncogenes^54^. Another study also showed that aberrant amplification of the 350–2000 kb genomic region containing the *MYCN* oncogene in neuroblastoma increased MYCN levels^23^.

### 3D structural changes produce oncogenic SE formation

Mammalian genomes are partitioned into a series of topologically associating domains (TADs) with an average size of approximately 1 Mb, and these TADs are the structural and functional units of chromosomes that function to spatially confine transcriptional regulatory circuits^29,59^. These TAD structures are invariant across diverse cell types and evolutionarily conserved in related species. Chromatin interactions are more frequent in TADs than they are outside TADs. It is now clear that TADs have the function of constraining long-range enhancer–promoter interactions, thereby insulating promoters from distal enhancers and SEs. Both genetic and epigenetic disruption of TAD boundaries allows new genes and enhancers/SEs to occupy spaces associated with enhancer/SE hijacking, altering regulatory contacts and leading to cancer^16,17,59^ (Fig. 2c).

TADs are formed by a chromatin loop architecture and are often involve the looping factors CCCTC-binding factor (CTCF) and cohesin. The presence of CTCF-associated boundary elements prevents ectopic contacts and insulates TADs from neighboring enhancers. Recently, a cis-expression structural alteration mapping algorithm was developed as a framework to systematically predict cancer-related gene overexpression. Through this approach, scientists have identified a TAD boundary deletion event that leads to the spreading of active chromatin to an adjacently fused TAD and generates an SE element, which can increase the expression of the *IRS4* gene in sarcoma and squamous cancer cells^53^. In another example, tandem duplications at the *IGF2* locus were found to extend over the intervening TAD boundary and to encompass an SE in colorectal cancer cells. This finding showed that the tandem duplication of *IGF2* and SE elements in the adjoining TAD led to de novo TAD formation and *IGF2* overexpression^53^. Furthermore, CTCF- and cohesion-binding sites acquire mutations in multiple cancer cell types. For example, CTCF- and cohesin-haploinsufficient mice are predisposed to cancer^60^. In T-ALL cells, CTCF-binding site disruption leads to the activation of *TAL1* and *LMO2* by regulatory elements outside of the insulated loops, resulting in T cell transformation^61^. In addition to mutations in TAD boundaries, epigenetic deregulation has also been demonstrated to be a mechanism for TAD disruption in gliomas^62^. It has been reported that increased methylation at the CTCF site and reduced CTCF binding result in the partial disruption of the TAD structure, which leads to the activation of PDGFRA (an oncogenic driver)^62^.

### Viral infection mediates SE formation

Virus infection induces SE formation to drive high-level transcription of some key genes involved in cell proliferation and survival. Viruses with oncogenic activity include Epstein–Barr virus (EBV), human papilloma virus (HPV), human T cell leukemia virus (HTLV), and human hepatitis B virus. After it infects human B cells, EBV produces oncoproteins, including EBNA2, 3A, 3C, and EBNALP. These oncoproteins activate NF-κB subunits and bind to SEs to drive the transcription of prosurvival and antiapoptotic genes such as *MYC, MIR155, IKZF3*, and *BCL2*, facilitating lymphoblastoid cell line growth^18,19^. Further studies showed that EBV SEs (ESEs) were transcribed into eRNAs, which facilitated the transcriptional activation of the *MYC* oncogene. Silencing MYC ESE eRNA inhibited the growth of cells^56^. A recent study indicated that the high-risk HPV oncoprotein E6 activates cervical cancer SEs to promote tumorigenesis by targeting the histone demethylase KDM5C^57^. Human lymphotropic virus type I (HTLV-I) frequently initiates adult T cell leukemia/lymphoma (ATLL). The proliferation of ATLL cells depends on BATF3 and IRF4, which cooperatively drive ATLL-specific gene expression. The viral transcription factor HBZ is expressed in all ATLL cases, and HBZ binds to the BATF3 SE and regulates the expression of the BATF3 and MYC genes, thereby contributing to ATLL cell proliferation^63^.

## Oncogenic SEs mediate the activation of signaling pathways and their mechanisms

Some oncogenic SEs activate several pathways, including Wnt^11,64,65^, TGF-β^11,66^, and LIF^11,67^, by regulating target genes. Moreover, oncogenic SEs are enriched in TF-binding sites that are associated with cancer signaling pathways^11^. These findings support the idea that SEs act as platforms for integrating regulatory signals that trigger target gene expression.

The Wnt pathway plays an important role in oncogenic SE mediation of tumorigenesis. A previous study showed that Wnt pathway-related SEs were enriched in binding sites for TCF4 (a terminal TF in the Wnt pathway) in colorectal cancer cells driven by the oncogenic Wnt pathway^11^. In a mouse model of basal cell carcinoma (BCC), a cell identity switch was enabled by a mostly permissive chromatin state accompanied by rapid Wnt pathway activation and reprogramming of the associated SEs. Treatment of BCC with Wnt pathway inhibitors reduced the residual tumor burden and enhanced cell differentiation^64^. Wnt signaling collaborates with chromatin architecture to post-transcriptionally dysregulate the expression of canonical cancer drivers. Recently obtained evidence has revealed that Wnt signaling and AHCTF1 promote oncogenic MYC expression through SE-mediated genes^65^. The cancer cell-specific gating of MYC is regulated by AHCTF1 (also known as ELYS), which connects nucleoporins to oncogenic SEs *via* β-catenin^65^.

In addition, TGF-β and LIF signaling also play vital roles in the development of cancers. TGF-β signaling is particularly important for increased tumor aggression and metastasis. In pancreatic cancer cells, the deletion of an SE in TGFBR2 significantly downregulated the expression of TGFBR2, resulting in impairment of the migration and EMT induced by TGF-β^66^. LIF was identified as an essential factor under the control of a cancer-specific SE. Osteosarcoma cells treated with a LIF recombinant protein displayed upregulated metastasis. UTX is a key activator of LIF transcription. GSK-J4, a UTX inhibitor, impaired SEs at the LIF gene locus, leading to LIF signaling pathway inhibition and subsequent defects 67.

In addition to the aforementioned signaling pathways, there are still many others that have important relationships with oncogenic SEs. Mutational RAS activity promotes oncogenic SE formation. The inhibition of aberrant RAS signaling results in the loss of active enhancer marks, SE decommissioning, and decreased gene expression^68^. In addition, RAS signaling can directly modulate SE function at enhancers by regulating the release of paused transcription. In rhabdomyosarcoma, for example, through the RAF–MEK–ERK MAPK pathway, oncogenic RAS inhibits myogenic differentiation by reducing MYOG expression, which is mediated by ERK2-dependent promoter-proximal stalling of RNA pol II at the MYOG locus. MEK inhibition with trametinib results in the loss of ERK2 at the MYOG promoter and the release of transcriptionally stalled MYOG expression, accompanied by the opening of chromatin and the establishment of SEs at myogenic-specific genes^69^.

## Therapeutic strategies targeting oncogenic SEs in cancer cells

Increasing evidence has revealed that SEs play vital roles in tumorigenesis, and oncogenic SEs could be promising therapeutic targets for cancer treatment. Inhibiting SE-driven oncogenic transcription is effective for therapy but presents significant challenges because transcription is a fundamental biological process common to all living cells^20^. Therefore, transcription inhibitors must selectively target oncogenic transcription while inducing only minimal toxicity in normal cells. Currently, there are two main kinds of small molecule inhibitors for targeting oncogenic SEs, BET inhibitors (BETis)^70^ and cyclin-dependent kinase (CDK) inhibitors^71^, which can selectively kill cancer cells by inhibiting the transcription of oncogenic SEs. The former are competitive inhibitors of bromodomain and extraterminal domain (BET) family proteins (BRD2, BRD3, BRD4, and BRDT), while the latter mainly target CDK7 and CDK9.

Transcription initiation, pausing, and elongation proceed through ordered activation of regulatory and enzymatic cofactors. Active oncogenic SEs enriched with H3K27ac marks can be recognized by BRD4, which interacts with the mediator coactivator complex, leading to the stepwise recruitment of CDK7 (a component of the TFIIH general transcription factor complex) and CDK9 (a component of P-TEFb, i.e., positive transcription elongation factor b)^72^. CDK7 is thought to primarily control transcriptional initiation by phosphorylating the RNA pol II C-terminal domain (CTD) at serine 5 (S5), serine 7 (S7), and TFIIE. CDK7 inhibition affects capping, pausing, elongation, and termination mostly through the phosphorylation of CTD and CDK9 in the activated T-loop. Ultimately, CDK7 facilitates the recruitment of the histone methyltransferases SETD1A/B and SETD2 through CTD phosphorylation and/or the activation of CDK9/P-TEFb^71^. CDK9 has been proven to control elongation. As a major CTD serine 2 (S2) kinase, CDK9 phosphorylates the NELF-E subunit of NELF and the SPT5 subunit of DSIF, allowing the release of RNA pol II that is paused at the proximal promoter to induce productive elongation^73^.

### Small molecule inhibitors targeting oncogenic SEs

Cancer cells hijack SEs to drive oncogene transcription, continuously promoting cell survival and proliferation. This aberrant SE-driven transcriptional event provides a new avenue for anticancer therapy. Thus, several small molecule inhibitors have been developed, and their potential preclinical effects *in vivo* and *in vitro* have been observed^47,74^. Some of them showed promising results in models established *in vitro* but have had largely disappointing results in clinical trials^75^. The first-generation CDK inhibitors, referred to as “pan-CDK” inhibitors, exhibited low affinity for CDKs and high cytotoxicity *in vivo*^75^. For example, flavopiridol, the most extensively investigated CDK inhibitor, can induce cell cycle arrest in the G1 and G2 phases in certain contexts and induce a cytotoxic response. Although it has broad-spectrum *in vitro* activity, it was less active *in vivo*. Tumor lysis syndrome was reported in approximately 40% of CLL patients treated with flavopiridol^76^. Thus, second-generation CDK inhibitors were developed with the aim of increased selectivity. BET inhibitors show a favorable activity profile, and hematologic (mainly thrombocytopenia) and nonhematologic adverse events (gastrointestinal toxicities, fatigue, bilirubin increase, etc.) are reversible upon treatment interruption^70^.

Previous studies have shown that SE-driven genes have a higher sensitivity to chromatin/transcriptional regulator inhibition than traditional enhancer-driven genes^11,23^. Treatment with the BETi JQ1 led to preferential loss of the BRD4 associated with SEs and consequent transcriptional elongation defects^21^. In other studies, heightened sensitivity was potentially attributed to at least two complementary mechanisms: (1) the cooperativity of constituent enhancers and (2) the short half-lives of oncogenic TFs^23,77^. SEs enriched with master TFs maintain TF expression *via* feedforward loops, and SE depletion may result in reduced transcription. In *MYCN*-amplified neuroblastoma, THZ1 treatment led to preferential downregulation of SE-associated genes, including *MYCN*, thus inhibiting the autoregulated suppression of MYCN-driven global transcription amplification^23^.

### BET inhibitors

Thienotriazolodiazepines were first characterized with antitumor activity and as inhibitors of acetylated histones that bind to bromodomain-containing proteins. A seminal report demonstrated that BETis could induce terminal differentiation and apoptosis in preclinical NUT (nuclear protein in testis) midline carcinoma models^78^. Chromosomal translocation, involving the NUT gene fusing to the BET gene BRD4, creates an in-frame BRD4–NUT oncogene, resulting in NUT midline carcinoma. Silencing of the BRD4–NUT fusion gene with BETis prevents the differentiation and proliferation of NUT carcinoma cells^78^. In preclinical models of AML and multiple myeloma, BETis (I-BET151 and JQ1) were reported to have a strong inhibitory effect on tumor progression^79,80^. Recent reports have also demonstrated that novel BETis have clear preclinical antitumor activity in a variety of solid tumors and hematologic cancers^81^. BETis target bromodomains and directly affect major transcription factors and key tissue- or cancer-specific genes, such as *MYC*^82^, *AR* and *TMPRSS2–ETS* fusion genes^83^, *TERT*^80^, *BCL2*^80^, *FOSL1*^84^, *E2F2*^85^, *ITK*^86^, *IL7R*^82^, *CDK6*^85^, *IRF4*^87^, and *IKZF1*^80,87^. In the BET family, BRD4 is considered to be an excellent target of BETis because of its important role in transcription. BETi-sensitive genes are associated with adjacent SEs. The TFs YAP and TAZ play crucial roles in the recruitment of BRD4 to SEs, and the enhancers/SEs occupied by YAP/TAZ show a preferential loss of BRD4 and sensitivity to JQ1 treatment^88^. Several BETi compounds (Table 1) have entered phase I or II clinical trials. Although these studies are still in the initial stage, they provide a new direction for the clinical treatment of cancers.

**Table 1:** Small molecule inhibitors targeting SE-driven transcription in tumors.

Target	Inhibitors	Disease	Mechanisms acting on related SE	Reference
BRD4	JQ1	DLBCL MM AML	Downregulation of SE-driven oncogenic/pathogenic and lineage-specific transcriptional circuits.	^21,22,96^
	OTX015/MK-8628	AML ALL GB NB DLBCL MM NMC	Downregulation of SE-driven oncogenes and other lineage-specific factors. Suppressing on NFkB/TLR/JAK/STAT signaling pathway genes and MYC- and E2F1-regulated genes.	^74,97–99^
	CPI-0610	MM Lymphoma	Downregulation of SE-associated and tumor addictive and lineage-specific gene.	^100^
	IBET-151	MM AML	Downregulation of SE-driven oncogenic and lineage-specific transcriptional circuits. Targeting BRD4-mediated RANKL-NF-kappa B signal pathway.	^101^
	BAY1238097	Solid tumors NHL	Downregulation of SE-associated and tumor addictive and lineage-specific gene.	^92,102^
CDK7	THZ1	ESCC NB ATCLL SCLC Melanoma TNBC	Downregulation of SE-associated and tumor addictive and lineage-specific gene, MYCN-driven transcriptional amplification.	^23,47,77,89,103,104^
	SY-1365	Ovarian cancer Breast cancer AML	Downregulation of SE-regulated oncogenes and other lineage-specific factors, enhanced in combination with the BCL2 inhibitor venetoclax.	^105^
CDK9	BI 894999	AML	Downregulation of SE-regulated oncogenes and other lineage-specific factors.	^106^
CDK8/19	Cortistatin A	AML	Upregulation of SE-associated genes linked to tumor suppression and lineage specification.	^92^
CDK12/13	THZ531	T-ALL ES	Downregulation of DNA damage response and SE-associated genes.	^93^
CDK4/6	LEE011	ES	Downregulation of SE-associated ES dependency genes CyclinD1/CDK4.	^107^

*Note*: ALL, acute lymphoblastic leukemia; AML, acute myeloid leukemia; ATCLL, adult T-cell leukemia/lymphoma; DLBCL, diffuse large B-cell lymphoma; ESCC, esophageal squamous cell carcinoma; ES, Ewing sarcoma; GB, glioblastoma; MM, multiple myeloma; NB, neuroblastoma; NHL, non-Hodgkin lymphomas; NMC, NUT-midline carcinoma; SCLC, small-cell lung cancer; T-ALL, T-cell acute lymphoblastic leukemia; TNBC, triple-negative breast cancer.

### Transcriptional CDK inhibitors

Historically, only three CDKs, CDK7, CDK9, and CDK8, were thought to be involved in the regulation of the transcription cycle. The discovery of small molecule inhibitors has provided another potential approach for targeting oncogenic SEs. In addition to pan-CDK inhibitors, there are many inhibitors selectively targeting CDK7 or CDK9, such as THZ1 and LDC067 (Table 1). THZ1 suppresses CDK7 by modifying cysteine 312^89^, with more than one-half of 1000 cancer cell lines showing IC50 values for THZ1 < 200 nM, leading to global transcriptional downregulation at high doses^6^. It was initially shown that sensitivity to THZ1 was conferred by targeting SE-driven RUNX1 in T-ALL^89^. Further reports revealed that THZ1 can selectively target SE-driven transcriptional processes in preclinical cell and mouse tumor models, such as those of triple-negative breast cancer (TNBC)^47^, MYCN-amplified neuroblastoma^23^, and small-cell lung cancer^77^. In AML preclinical models, CKIα inhibitors targeting CDK7 and CDK9 augment CKIα-induced p53 activation, suppress SE-driven oncogenes, and induce apoptosis^90^. LDC067 is one of the first compounds found to have CDK9 selectivity, and it has been proven to have 55–230-fold greater selectivity for CDK9 than for the other CDKs^91^. Treatment with LDC067 can inhibit transcription and induce apoptosis in HeLa cells and primary AML blasts^91^. CDK8 has been implicated in the transcription driven by SE-controlled genes. Cortistatin A exhibits high affinity for CDK8 and CDK19 and has antiproliferative activity against multiple leukemia cell lines *in vitro* and in AML mouse models^92^. THZ531, a CDK12/13 inhibitor, synergizes with PARP inhibitors in models of Ewing sarcoma *in vitro* and *in vivo*^93^. Although structural and biological validation of these inhibitors has not been completed, their suppression of SE-associated transcriptional regulators provides a novel approach for targeting oncogenes.

## Concluding remarks

SEs play vital roles in transcriptional regulation and have pathogenic ability, especially oncogenic ability, in a context-dependent manner. Despite compelling evidence that SEs regulate cell identity genes, there is still no clear understanding of how SEs play regulatory roles. NGS technology recently provided a new means of mapping the genomic landscape in greater detail. ChIP-seq data and bioinformatics algorithms are being utilized to identify genomic proximity and assign SEs to target genes. However, knowledge of the intrinsic structure of SEs and of their interactions with target genes in three-dimensional space is still lacking; therefore, a comprehensive approach involving 5C and functional screening is needed. Other new technologies, including Hi-C, ATAC-seq, Hi-ChIP, CUT&Tag, the CRISPR genome-editing tool, and single-cell sequencing technology, are also being used to reveal the mechanisms of SEs that regulate transcription and oncogenesis. From a therapeutic standpoint, the discovery of SE targeting by JQ1 led to the development of first- and second-generation BET inhibitors. Since BETs were discovered, small molecule inhibitors targeting individual SE components have shown great promise for clinical application. However, resistance to single-agent treatments of BETis^94^ and THZ1^95^ has been reported in many preclinical models. Therefore, future exploration of SEs will focus on clarifying how SE components regulate SE function and how to better utilize SEs in targeted therapy.

## References

[1] Banerji J, Rusconi S, Schaffner W (1981). Expression of a beta-globin gene is enhanced by remote SV40 DNA sequences. Cell.

[2] Spitz F, Furlong EEM (2012). Transcription factors: from enhancer binding to developmental control. Nat. Rev. Genet..

[3] Kundaje A (2015). Integrative analysis of 111 reference human epigenomes. Nature.

[4] Leveille N (2015). Genome-wide profiling of p53-regulated enhancer RNAs uncovers a subset of enhancers controlled by a lncRNA. Nat. Commun..

[5] Whyte WA (2013). Master transcription factors and mediator establish super-enhancers at key cell identity genes. Cell.

[6] Hnisz D (2013). Super-enhancers in the control of cell identity and disease. Cell.

[7] Hah N (2015). Inflammation-sensitive super enhancers form domains of coordinately regulated enhancer RNAs. Proc. Natl. Acad. Sci. U.S.A..

[8] Santos-Pereira JM, Aguilera A (2015). R loops: new modulators of genome dynamics and function. Nat. Rev. Genet..

[9] Shin HY (2016). Hierarchy within the mammary STAT5-driven Wap super-enhancer. Nat. Genet..

[10] Hay D (2016). Genetic dissection of the alpha-globin super-enhancer in vivo. Nat. Genet..

[11] Hnisz D (2015). Convergence of developmental and oncogenic signaling pathways at transcriptional super-enhancers. Mol. Cell.

[12] Mansour MR (2014). Oncogene regulation. An oncogenic super-enhancer formed through somatic mutation of a noncoding intergenic element. Science.

[13] Krijger PH, de Laat W (2016). Regulation of disease-associated gene expression in the 3D genome. Nat. Rev. Mol. Cell Biol..

[14] Valentijn LJ (2015). TERT rearrangements are frequent in neuroblastoma and identify aggressive tumors. Nat. Genet..

[15] Groschel S (2014). A single oncogenic enhancer rearrangement causes concomitant EVI1 and GATA2 deregulation in leukemia. Cell.

[16] Spielmann M, Lupiáñez DG, Mundlos S (2018). Structural variation in the 3D genome. Nat. Rev. Genet..

[17] Furlong EEM, Levine M (2018). Developmental enhancers and chromosome topology. Science.

[18] Gunnell A (2016). RUNX super-enhancer control through the Notch pathway by Epstein–Barr virus transcription factors regulates B cell growth. Nucleic Acids Res..

[19] Zhou H (2015). Epstein–Barr virus oncoprotein super-enhancers control B cell growth. Cell Host Microbe.

[20] Bradner JE, Hnisz D, Young RA (2017). Transcriptional addiction in cancer. Cell.

[21] Loven J (2013). Selective inhibition of tumor oncogenes by disruption of super-enhancers. Cell.

[22] Chapuy B (2013). Discovery and characterization of super-enhancer-associated dependencies in diffuse large B cell lymphoma. Cancer Cell.

[23] Chipumuro E (2014). CDK7 inhibition suppresses super-enhancer-linked oncogenic transcription in MYCN-driven cancer. Cell.

[24] Hnisz D, Shrinivas K, Young RA, Chakraborty AK, Sharp PA (2017). A phase separation model for transcriptional control. Cell.

[25] Sabari BR (2018). Coactivator condensation at super-enhancers links phase separation and gene control. Science.

[26] Strom AR (2017). Phase separation drives heterochromatin domain formation. Nature.

[27] Lin Y, Protter DS, Rosen MK, Parker R (2015). Formation and maturation of phase-separated liquid droplets by RNA-binding proteins. Mol. Cell.

[28] Boija A (2018). Transcription factors activate genes through the phase-separation capacity of their activation domains. Cell.

[29] Rao SSP (2017). Cohesin loss eliminates all loop domains. Cell.

[30] Visel A (2009). ChIP-seq accurately predicts tissue-specific activity of enhancers. Nature.

[31] Boyle AP (2008). High-resolution mapping and characterization of open chromatin across the genome. Cell.

[32] Jin F (2013). A high-resolution map of the three-dimensional chromatin interactome in human cells. Nature.

[33] Rhee HS, Pugh BF (2011). Comprehensive genome-wide protein–DNA interactions detected at single-nucleotide resolution. Cell.

[34] Bell O, Tiwari VK, Thoma NH, Schubeler D (2011). Determinants and dynamics of genome accessibility. Nat. Rev. Genet..

[35] Kim TK (2010). Widespread transcription at neuronal activity-regulated enhancers. Nature.

[36] Fullwood MJ (2009). An oestrogen-receptor-alpha-bound human chromatin interactome. Nature.

[37] Zhao X (2019). BCL2 amplicon loss and transcriptional remodeling drives ABT-199 resistance in B cell lymphoma models. Cancer Cell.

[38] Arnold CD (2013). Genome-wide quantitative enhancer activity maps identified by STARR-seq. Science.

[39] Mifsud B (2015). Mapping long-range promoter contacts in human cells with high-resolution capture Hi-C. Nat. Genet..

[40] Mumbach MR (2016). HiChIP: efficient and sensitive analysis of protein-directed genome architecture. Nat. Methods.

[41] Northcott PA (2014). Enhancer hijacking activates GFI1 family oncogenes in medulloblastoma. Nature.

[42] Sengupta S, George RE (2017). Super-enhancer-driven transcriptional dependencies in cancer. Trends Cancer.

[43] Nakamura Y (2017). Targeting of super-enhancers and mutant BRAF can suppress growth of BRAF -mutant colon cancer cells via repression of MAPK signaling pathway. Cancer Lett..

[44] Babu D, Fullwood MJ (2017). Expanding the effects of ERG on chromatin landscapes and dysregulated transcription in prostate cancer. Nat. Genet..

[45] Shen Y (2017). Recombinant decorin fusion protein attenuates murine abdominal aortic aneurysm formation and rupture. Sci. Rep..

[46] Dwight T (2018). TERT structural rearrangements in metastatic pheochromocytomas. Endocr.-Relat. Cancer.

[47] Wang Y (2015). CDK7-dependent transcriptional addiction in triple-negative breast cancer. Cell.

[48] Schaub MA, Boyle AP, Kundaje A, Batzoglou S, Snyder M (2012). Linking disease associations with regulatory information in the human genome. Genome Res..

[49] Maurano MT (2012). Systematic localization of common disease-associated variation in regulatory DNA. Science.

[50] Zhang X (2019). BRCA1 mutations attenuate super-enhancer function and chromatin looping in haploinsufficient human breast epithelial cells. Breast Cancer Res..

[51] Kandaswamy R (2016). Genetic predisposition to chronic lymphocytic leukemia is mediated by a BMF super-enhancer polymorphism. Cell Rep..

[52] Oldridge DA (2015). Genetic predisposition to neuroblastoma mediated by a LMO1 super-enhancer polymorphism. Nature.

[53] Weischenfeldt J (2017). Pan-cancer analysis of somatic copy-number alterations implicates IRS4 and IGF2 in enhancer hijacking. Nat. Genet..

[54] Zhang X (2016). Identification of focally amplified lineage-specific super-enhancers in human epithelial cancers. Nat. Genet..

[55] Drier Y (2016). An oncogenic MYB feedback loop drives alternate cell fates in adenoid cystic carcinoma. Nat. Genet..

[56] Liang J (2016). Epstein–Barr virus super-enhancer eRNAs are essential for MYC oncogene expression and lymphoblast proliferation. Proc. Natl. Acad. Sci. U.S.A..

[57] Chen X (2018). E6 protein expressed by high-risk HPV activates super-enhancers of the EGFR and c-MET oncogenes by destabilizing the histone demethylase KDM5C. Cancer Res..

[58] Sin-Chan P (2019). A C19MC-LIN28A-MYCN oncogenic circuit driven by hijacked super-enhancers is a distinct therapeutic vulnerability in ETMRs: a lethal brain tumor. Cancer Cell.

[59] Dixon JR (2012). Topological domains in mammalian genomes identified by analysis of chromatin interactions. Nature.

[60] Viny AD (2015). Dose-dependent role of the cohesin complex in normal and malignant hematopoiesis. J. Exp. Med..

[61] Hnisz D (2016). Activation of proto-oncogenes by disruption of chromosome neighborhoods. Science.

[62] Flavahan WA (2016). Insulator dysfunction and oncogene activation in IDH mutant gliomas. Nature.

[63] Nakagawa M (2018). Targeting the HTLV-I-regulated BATF3/IRF4 transcriptional network in adult T cell leukemia/lymphoma. Cancer Cell.

[64] Biehs B (2018). A cell identity switch allows residual BCC to survive Hedgehog pathway inhibition. Nature.

[65] Scholz BA (2019). WNT signaling and AHCTF1 promote oncogenic MYC expression through super-enhancer-mediated gene gating. Nat. Genet..

[66] Zhu X (2019). A super-enhancer controls TGF-beta signaling in pancreatic cancer through downregulation of TGFBR2. Cell Signal..

[67] Lu B (2019). Epigenetic profiling identifies LIF as a super-enhancer-controlled regulator of stem cell-like properties in osteosarcoma. Mol. Cancer Res..

[68] Nabet B (2015). Deregulation of the Ras-Erk signaling axis modulates the enhancer landscape. Cell Rep..

[69] Yohe ME (2018). MEK inhibition induces MYOG and remodels super-enhancers in RAS-driven rhabdomyosarcoma. Sci. Trans. Med..

[70] Stathis A, Bertoni F (2018). BET proteins as targets for anticancer treatment. Cancer Discov..

[71] Galbraith MD, Bender H, Espinosa JM (2019). Therapeutic targeting of transcriptional cyclin-dependent kinases. Transcription.

[72] Hajmirza A (2018). BET family protein BRD4: an emerging actor in NFkappaB signaling in inflammation and cancer. Biomedicines.

[73] Zhou Q, Li T, Price DH (2012). RNA polymerase II elongation control. Annu. Rev. Biochem..

[74] Berthon C (2016). Bromodomain inhibitor OTX015 in patients with acute leukaemia: a dose-escalation, phase 1 study. Lancet Haematol..

[75] Asghar U, Witkiewicz AK, Turner NC, Knudsen ES (2015). The history and future of targeting cyclin-dependent kinases in cancer therapy. Nat. Rev. Drug Discov..

[76] Bose P, Simmons GL, Grant S (2013). Cyclin-dependent kinase inhibitor therapy for hematologic malignancies. Expert Opin. Investig. Drugs.

[77] Christensen CL (2014). Targeting transcriptional addictions in small cell lung cancer with a covalent CDK7 inhibitor. Cancer Cell.

[78] Filippakopoulos P (2010). Selective inhibition of BET bromodomains. Nature.

[79] Zuber J (2011). RNAi screen identifies Brd4 as a therapeutic target in acute myeloid leukaemia. Nature.

[80] Delmore JE (2011). BET bromodomain inhibition as a therapeutic strategy to target c-Myc. Cell.

[81] Ali I, Choi G, Lee K (2017). BET inhibitors as anticancer agents: a patent review. Recent Pat. Anti-Cancer Drug Discov..

[82] Bernasconi E (2017). Preclinical evaluation of the BET bromodomain inhibitor BAY 1238097 for the treatment of lymphoma. Br. J. Haematol..

[83] Faivre EJ (2017). Exploitation of castration-resistant prostate cancer transcription factor dependencies by the novel BET inhibitor ABBV-075. Mol. Cancer Res..

[84] Baker EK (2015). BET inhibitors induce apoptosis through a MYC independent mechanism and synergise with CDK inhibitors to kill osteosarcoma cells. Sci. Rep..

[85] Riveiro ME (2016). OTX015 (MK-8628), a novel BET inhibitor, exhibits antitumor activity in non-small cell and small cell lung cancer models harboring different oncogenic mutations. Oncotarget.

[86] Rhyasen GW (2016). AZD5153: a novel bivalent BET bromodomain inhibitor highly active against hematologic malignancies. Mol. Cancer Ther..

[87] Siu KT (2017). Preclinical activity of CPI-0610, a novel small-molecule bromodomain and extra-terminal protein inhibitor in the therapy of multiple myeloma. Leukemia.

[88] Zanconato F (2018). Transcriptional addiction in cancer cells is mediated by YAP/TAZ through BRD4. Nat. Med..

[89] Kwiatkowski N (2014). Targeting transcription regulation in cancer with a covalent CDK7 inhibitor. Nature.

[90] Minzel W (2018). Small molecules co-targeting CKIalpha and the transcriptional kinases CDK7/9 control AML in preclinical models. Cell.

[91] Albert TK (2014). Characterization of molecular and cellular functions of the cyclin-dependent kinase CDK9 using a novel specific inhibitor. Br. J. Pharm..

[92] Pelish HE (2015). Mediator kinase inhibition further activates super-enhancer-associated genes in AML. Nature.

[93] Iniguez AB (2018). EWS/FLI confers tumor cell synthetic lethality to CDK12 inhibition in Ewing sarcoma. Cancer Cell.

[94] Fong CY (2015). BET inhibitor resistance emerges from leukaemia stem cells. Nature.

[95] Gao Y (2018). Overcoming resistance to the THZ series of covalent transcriptional CDK inhibitors. Cell Chem. Biol..

[96] Bhagwat AS (2016). BET bromodomain inhibition releases the mediator complex from select cis-regulatory elements. Cell Rep..

[97] Hottinger AF (2016). Dose optimization of MK-8628 (OTX015), a small molecule inhibitor of bromodomain and extra-terminal (BET) proteins, in patients (pts) with recurrent glioblastoma (GB). J. Clin. Oncol..

[98] Henssen A (2016). Targeting MYCN-driven transcription by BET-bromodomain inhibition. Clin. Cancer Res..

[99] Boi M (2015). The BET bromodomain inhibitor OTX015 affects pathogenetic pathways in preclinical B-cell tumor models and synergizes with targeted drugs. Clin. Cancer Res..

[100] Abramson JS (2015). BET inhibitor CPI-0610 is well tolerated and induces responses in diffuse large B-cell lymphoma and follicular lymphoma: preliminary analysis of an ongoing Phase 1 study. Blood.

[101] Guo NH, Zheng JF, Zi FM, Cheng J (2019). I-BET151 suppresses osteoclast formation and inflammatory cytokines secretion by targetting BRD4 in multiple myeloma. Biosci. Rep..

[102] Postel-Vinay S (2016). First-in-human phase I dose escalation study of the Bromodomain and Extra-Terminal motif (BET) inhibitor BAY 1238097 in subjects with advanced malignancies. Eur. J. Cancer.

[103] Eliades P (2018). High MITF expression is associated with super-enhancers and suppressed by CDK7 inhibition in melanoma. J. Investig. Dermatol..

[104] Jiang YY (2017). Targeting super-enhancer-associated oncogenes in oesophageal squamous cell carcinoma. Gut.

[105] Hu S (2019). Discovery and characterization of SY-1365, a selective, covalent inhibitor of CDK7. Cancer Res..

[106] Gerlach D (2018). The novel BET bromodomain inhibitor BI 894999 represses super-enhancer-associated transcription and synergizes with CDK9 inhibition in AML. Oncogene.

[107] Kennedy AL (2015). Functional, chemical genomic, and super-enhancer screening identify sensitivity to cyclin D1/CDK4 pathway inhibition in Ewing sarcoma. Oncotarget.

